# An ultrapotent synthetic nanobody neutralizes SARS-CoV-2 by stabilizing inactive Spike

**DOI:** 10.1126/science.abe3255

**Published:** 2020-11-05

**Authors:** Michael Schoof, Bryan Faust, Reuben A. Saunders, Smriti Sangwan, Veronica Rezelj, Nick Hoppe, Morgane Boone, Christian B. Billesbølle, Cristina Puchades, Caleigh M. Azumaya, Huong T. Kratochvil, Marcell Zimanyi, Ishan Deshpande, Jiahao Liang, Sasha Dickinson, Henry C. Nguyen, Cynthia M. Chio, Gregory E. Merz, Michael C. Thompson, Devan Diwanji, Kaitlin Schaefer, Aditya A. Anand, Niv Dobzinski, Beth Shoshana Zha, Camille R. Simoneau, Kristoffer Leon, Kris M. White, Un Seng Chio, Meghna Gupta, Mingliang Jin, Fei Li, Yanxin Liu, Kaihua Zhang, David Bulkley, Ming Sun, Amber M. Smith, Alexandrea N. Rizo, Frank Moss, Axel F. Brilot, Sergei Pourmal, Raphael Trenker, Thomas Pospiech, Sayan Gupta, Benjamin Barsi-Rhyne, Vladislav Belyy, Andrew W. Barile-Hill, Silke Nock, Yuwei Liu, Nevan J. Krogan, Corie Y. Ralston, Danielle L. Swaney, Adolfo García-Sastre, Melanie Ott, Marco Vignuzzi, QCRG Structural Biology Consortium, Peter Walter, Aashish Manglik

**Affiliations:** 1 Howard Hughes Medical Institute, University of California at San Francisco, San Francisco, CA, USA.; 2 Department of Biochemistry and Biophysics, University of California at San Francisco, San Francisco, CA, USA.; 3 Department of Pharmaceutical Chemistry, University of California at San Francisco, San Francisco, CA, USA.; 4 Quantitative Biosciences Institute (QBI) Coronavirus Research Group Structural Biology Consortium, University of California, San Francisco, CA, USA.; 5 Department of Cellular and Molecular Pharmacology, University of California at San Francisco, San Francisco, CA, USA.; 6 Viral Populations and Pathogenesis Unit, CNRS UMR 3569, Institut Pasteur, 75724 Paris Cedex 15, France.; 7 Department of Pulmonary, Critical Care, Allergy and Sleep Medicine, University of California San Francisco, San Francisco, CA, USA.; 8 Quantitative Biosciences Institute (QBI), University of California San Francisco, San Francisco, CA, USA.; 9 J. David Gladstone Institutes, San Francisco, CA, USA.; 10 Department of Medicine, University of California San Francisco, San Francisco, CA, USA.; 11 Department of Microbiology, Icahn School of Medicine at Mount Sinai, New York, NY, USA.; 12 Global Health and Emerging Pathogens Institute, Icahn School of Medicine at Mount Sinai, New York, NY, USA.; 13 Molecular Biophysics and Integrated Bioimaging and the Molecular Foundry, Lawrence Berkeley National Laboratory, Berkeley, CA, USA.; 14 Cytiva Life Sciences, Marlborough, MA, USA.; 15 Department of Medicine, Division of Infectious Diseases, Icahn School of Medicine at Mount Sinai, New York, NY, USA.; 16 The Tisch Cancer Institute, Icahn School of Medicine at Mount Sinai, New York, NY, USA.; 17 Department of Anesthesia and Perioperative Care, University of California at San Francisco, San Francisco, CA, USA.

## Abstract

The severe acute respiratory syndrome coronavirus 2 (SARS-CoV-2) virus enters host cells via an interaction between its Spike protein and the host cell receptor angiotensin-converting enzyme 2 (ACE2). By screening a yeast surface-displayed library of synthetic nanobody sequences, we developed nanobodies that disrupt the interaction between Spike and ACE2. Cryo–electron microscopy (cryo-EM) revealed that one nanobody, Nb6, binds Spike in a fully inactive conformation with its receptor binding domains locked into their inaccessible down state, incapable of binding ACE2. Affinity maturation and structure-guided design of multivalency yielded a trivalent nanobody, mNb6-tri, with femtomolar affinity for Spike and picomolar neutralization of SARS-CoV-2 infection. mNb6-tri retains function after aerosolization, lyophilization, and heat treatment, which enables aerosol-mediated delivery of this potent neutralizer directly to the airway epithelia.

## Nanobodies that neutralize

Monoclonal antibodies that bind to the spike protein of severe acute respiratory syndrome coronavirus 2 (SARS-CoV-2) show therapeutic promise but must be produced in mammalian cells and need to be delivered intravenously. By contrast, single-domain antibodies called nanobodies can be produced in bacteria or yeast, and their stability may enable aerosol delivery. Two papers now report nanobodies that bind tightly to spike and efficiently neutralize SARS-CoV-2 in cells. Schoof *et al.* screened a yeast surface display of synthetic nanobodies and Xiang *et al.* screened anti-spike nanobodies produced by a llama. Both groups identified highly potent nanobodies that lock the spike protein in an inactive conformation. Multivalent constructs of selected nanobodies achieved even more potent neutralization.

*Science*, this issue p. 1473, p. 1479

## Teaser

Potent neutralizers of SARS-CoV-2 are identified by screening either synthetic or llama-produced nanobodies.

Over the past two decades, three zoonotic β-coronaviruses have entered the human population, causing severe respiratory symptoms with high mortality (*1*–*3*). The COVID-19 pandemic is caused by severe acute respiratory syndrome coronavirus 2 (SARS-CoV-2), the most readily transmissible of these three coronaviruses (*4*–*7*). No preventive treatment has been approved for any coronavirus to date, and the timeline for an effective and broadly available vaccine for SARS-CoV-2 remains uncertain. The development of new therapeutic and prophylactic approaches thus remains essential.

Coronavirus virions are bounded by a membrane that contains the homotrimeric transmembrane glycoprotein Spike, which is responsible for virus entry into the host cell (*8*, *9*). The surface-exposed portion of Spike is composed of two domains, S_1_ and S_2_ (*10*). S_1_ binds the host cell receptor angiotensin-converting enzyme 2 (ACE2), whereas S_2_ catalyzes fusion of the viral and host cell membranes (*11*–*13*). Contained within S_1_ is the receptor binding domain (RBD), which directly binds to ACE2, and the N-terminal domain (NTD). The RBD is attached to the body of Spike by a flexible region and can exist in an inaccessible down state or an accessible up state (*14*, *15*). Binding to ACE2 requires the RBD to occupy the up state and enables cleavage by host proteases, triggering a conformational change in S_2_ required for viral entry (*16*). In SARS-CoV-2 virions, Spike exchanges between an active, open conformation with at least one RBD in the up state and an inactive, closed conformation with all RBDs in the down state (*8*, *9*).

**Fig. 1:**
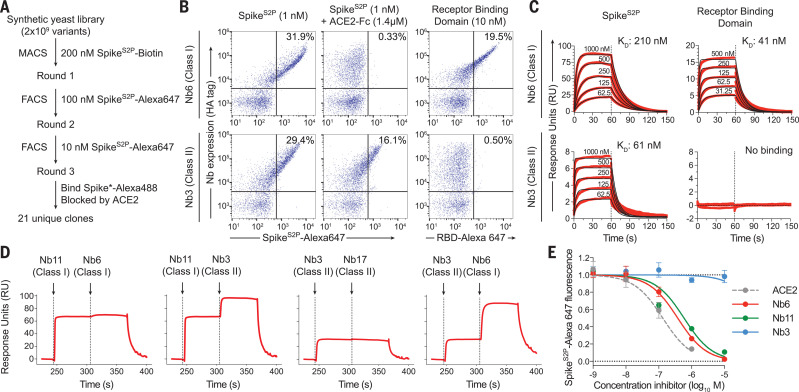
Discovery of two distinct classes of anti-Spike nanobodies. (**A**) Selection strategy for identification of anti-Spike nanobodies that disrupt Spike-ACE2 interactions using magnetic bead selections (MACS) or fluorescence-activated cell sorting (FACS). (**B**) Flow cytometry of yeast displaying Nb6 (a class I nanobody) or Nb3 (a class II nanobody). Nb6 binds Spike^S2P^-Alexa 647 and the RBD (RBD-Alexa 647). Nb6 binding to Spike^S2P^ is completely disrupted by an excess (1.4 μM) of ACE2-Fc. Nb3 binds Spike^S2P^ but not the RBD. Nb3 binding to Spike^S2P^ is partially decreased by ACE2-Fc. (**C**) SPR of Nb6 and Nb3 binding to either Spike^S2P^ or RBD. Red traces are raw data, and global kinetic fits are shown in black. Nb3 shows no binding to RBD. (**D**) SPR experiments with immobilized Spike^S2P^ show that class I and class II nanobodies can bind Spike^S2P^ simultaneously. By contrast, two class I nanobodies or class II nanobodies do not bind simultaneously. (**E**) Nanobody inhibition of 1 nM Spike^S2P^-Alexa 647 binding to ACE2-expressing HEK293T cells. *n* = 3 (ACE2, Nb3) or *n* = 5 (Nb6, Nb11) biological replicates. All error bars represent SEM.

We isolated single-domain antibodies (nanobodies) that neutralize SARS-CoV-2 by screening a yeast surface-displayed library of >2 × 10^9^ synthetic nanobody sequences for binders to the Spike ectodomain (*17*). We used a mutant form of SARS-CoV-2 Spike (Spike^S2P^) as the antigen (*15*). Spike^S2P^ lacks one of the two proteolytic cleavage sites between the S_1_ and S_2_ domains and introduces two mutations and a trimerization domain to stabilize the prefusion conformation. We labeled Spike^S2P^ with biotin or with fluorescent dyes and selected nanobody-displaying yeast over multiple rounds, first by magnetic bead binding and then by fluorescence-activated cell sorting (Fig. 1A).

Three rounds of selection yielded 21 distinct nanobodies that bound Spike^S2P^ and showed decreased binding in the presence of a dimeric construct of the ACE2 extracellular domain (ACE2-Fc). These nanobodies fall into two classes. Class I binds the RBD and competes directly with ACE2-Fc (Fig. 1B). A prototypical example of this class is nanobody Nb6, which binds to Spike^S2P^ and to RBD alone with a dissociation constant (*K*_D_) of 210 and 41 nM, respectively (Fig. 1C and table S1). Class II, exemplified by nanobody Nb3, binds to Spike^S2P^ (*K*_D_ = 61 nM) but displays no binding to RBD alone (Fig. 1C and table S1). In the presence of excess ACE2-Fc, binding of Nb6 and other class I nanobodies is blocked entirely, whereas binding of Nb3 and other class II nanobodies is moderately decreased (Fig. 1B). These results suggest that class I nanobodies target the RBD to block ACE2 binding, whereas class II nanobodies target other epitopes. Indeed, surface plasmon resonance (SPR) experiments demonstrate that class I and class II nanobodies can bind Spike^S2P^ simultaneously (Fig. 1D).

Class I nanobodies show a consistently faster association rate constant (*k*_a_) for nanobody binding to the isolated RBD than to Spike^S2P^ (table S1), which suggests that RBD accessibility influences the *K*_D_. We next tested the efficacy of class I and class II nanobodies to inhibit binding of fluorescently labeled Spike^S2P^ to ACE2-expressing human embryonic kidney (HEK) 293 cells (Fig. 1E and table S1). Class I nanobodies Nb6 and Nb11 emerged as two of the most potent clones, with half-maximal inhibitory concentration (IC_50_) values of 370 and 540 nM, respectively. Class II nanobodies showed little to no activity in this assay. We prioritized two class I nanobodies, Nb6 and Nb11, that combine potent Spike^S2P^ binding with relatively small differences in *k*_a_ between binding to Spike^S2P^ or RBD. For class II nanobodies, we prioritized Nb3 because of its relative yield during purification (table S1).

**Fig. 2:**
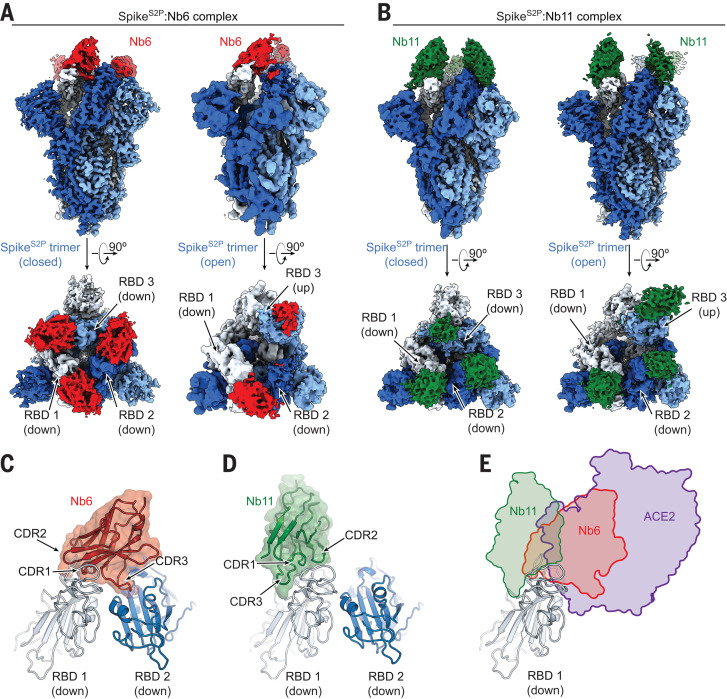
Cryo-EM structures of Nb6 and Nb11 bound to Spike. (**A**) Cryo-EM maps of the Spike^S2P^-Nb6 complex in either closed (left) or open (right) Spike^S2P^ conformation. (**B**) Cryo-EM maps of the Spike^S2P^-Nb11 complex in either closed (left) or open (right) Spike^S2P^ conformation. The top views show RBD up or down states. (**C**) Nb6 straddles the interface of two down-state RBDs, with CDR3 reaching over to an adjacent RBD. (**D**) Nb11 binds a single RBD in the down state (displayed) or similarly in the up state. No cross-RBD contacts are made by Nb11 in either RBD up or down state. (**E**) Comparison of RBD epitopes engaged by ACE2 (purple), Nb6 (red), or Nb11 (green). Both Nb11 and Nb6 directly compete with ACE2 binding.

To define the binding sites of Nb6 and Nb11, we determined their cryo–electron microscopy (cryo-EM) structures bound to Spike^S2P^ (Fig. 2, A and B; figs. S1 to S3; and table S2). Both nanobodies recognize RBD epitopes that overlap the ACE2 binding site (Fig. 2E). For Nb6 and Nb11, we resolved nanobody binding to both the open and closed conformations of Spike^S2P^. We obtained a 3.0-Å map of Nb6 bound to closed Spike^S2P^, which enabled modeling of the Nb6-Spike^S2P^ complex (Fig. 2A), including the complementarity-determining regions (CDRs). We also obtained lower-resolution maps for Nb6 bound to open Spike^S2P^ (3.8 Å), and Nb11 bound to open and closed Spike^S2P^ (4.2 and 3.7 Å, respectively). For these lower-resolution maps, we could define the nanobody’s binding orientation but not accurately model the CDRs.

Nb6 bound to closed Spike^S2P^ straddles the interface between two adjacent RBDs. Most of the contacting surfaces are contributed by CDR1 and CDR2 of Nb6 (Fig. 2C). CDR3 contacts the adjacent RBD positioned counterclockwise when viewed from the top (Fig. 2C). The binding of one Nb6 therefore stabilizes two adjacent RBDs in the down state and likely preorganizes the binding site for a second and third Nb6 molecule to stabilize the closed Spike conformation. By contrast, Nb11 bound to down-state RBDs only contacts a single RBD (Fig. 2D).

The structure of Nb6 bound to closed Spike^S2P^ enabled us to engineer bivalent and trivalent nanobodies predicted to lock all RBDs in the down state. We inserted flexible Gly-Ser linkers of either 15 or 20 amino acids to span the 52-Å distance between adjacent Nb6 monomers bound to down-state RBDs in closed Spike^S2P^ (fig. S4). These linkers are too short to span the 72-Å distance between Nb6 molecules bound to open Spike. Moreover, steric clashes would prevent binding of three RBDs in open Spike with a single up-state RBD even with longer linker length (fig. S4). By contrast, the minimum distance between adjacent Nb11 monomers bound to either open or closed Spike^S2P^ is 68 Å. We predicted that multivalent binding by Nb6 constructs would display substantially slowed dissociation rates owing to enhanced avidity.

**Fig. 3:**
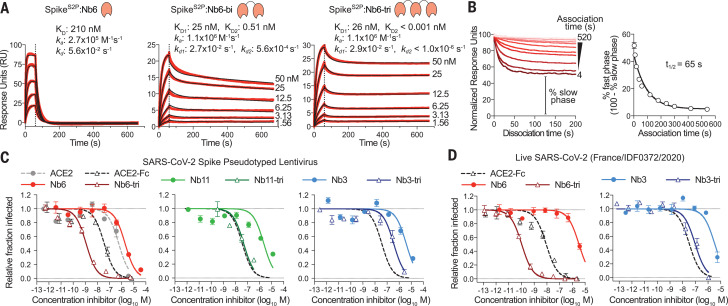
Multivalency improves nanobody affinity and inhibitory efficacy. (**A**) SPR of Nb6 and multivalent variants. Red traces show raw data, and black lines show global kinetic fit for Nb6 and independent fits for association and dissociation phases for Nb6-bi and Nb6-tri. (**B**) Dissociation phase SPR traces for Nb6-tri after variable association times ranging from 4 to 520 s. Curves were normalized to maximal signal at the beginning of the dissociation phase. Percent fast-phase dissociation is plotted as a function of association time (right) with a single exponential fit. *n* = 3 independent biological replicates. (**C**) Inhibition of pseudotyped lentivirus infection of ACE2-expressing HEK293T cells. *n* = 3 biological replicates for all but Nb11-tri (*n* = 2). (**D**) Inhibition of live SARS-CoV-2 virus. Representative biological replicate with *n* = 3 (right) or *n* = 4 (left) technical replicates per concentration. *n* = 3 biological replicates for all but Nb3 and Nb3-tri (*n* = 2). All error bars represent SEM.

In SPR experiments, both bivalent Nb6 with a 15–amino acid linker (Nb6-bi) and trivalent Nb6 with two 20–amino acid linkers (Nb6-tri) dissociate from Spike^S2P^ in a biphasic manner. The dissociation phase can be fitted to two components: a fast phase with kinetic rate constants *k*_d1_ of 2.7 × 10^−2^ s^−1^ for Nb6-bi and 2.9 × 10^−2^ s^−1^ for Nb6-tri, which are close to that observed for monovalent Nb6 (*k*_d_ = 5.6 × 10^−2^ s^−1^), and a slow phase that is dependent on avidity (*k*_d2_ = 3.1 × 10^−4^ s^−1^ for Nb6-bi and *k*_d2_ < 1.0 × 10^−6^ s^−1^ for Nb6-tri) (Fig. 3A). The relatively similar *k*_d_ for the fast phase suggests that a fraction of the observed binding for the multivalent constructs is nanobody binding to a single Spike^S2P^ RBD. By contrast, the slow dissociation phase of Nb6-bi and Nb6-tri indicates engagement of two or three RBDs. We observed no dissociation for the slow phase of Nb6-tri over 10 min, indicating an upper boundary for *k*_d2_ of 1 × 10^−6^ s^−1^ and subpicomolar affinity. This measurement remains an upper boundary estimate because the measurement is limited by the intrinsic dissociation rate of Spike^S2P^ from the SPR chip imposed by the chemistry used to immobilize Spike^S2P^. The true dissociation rate, therefore, may be considerably lower.

Biphasic dissociation could be explained by a slow interconversion between up- and down-state RBDs, with conversion to the more stable down state required for multivalent binding: A single domain of Nb6-tri engaged with an up-state RBD would dissociate rapidly. The system would then reequilibrate as the RBD flips into the down state, eventually allowing Nb6-tri to trap all RBDs in closed Spike^S2P^. To test this directly, we varied the association time for Nb6-tri binding to Spike^S2P^. Indeed, we observed an exponential decrease in the percentage of fast-phase dissociation with a half-life (*t*_1/2_) of 65 s (Fig. 3B), which, we surmise, reflects the time scale of conversion between the RBD up and down states in Spike^S2P^. Taken together, dimerization and trimerization of Nb6 afforded 750-fold and >200,000-fold gains in *K*_D_, respectively.

Unable to determine the binding site of Nb3 by cryo-EM, we turned to radiolytic hydroxyl radical footprinting. We exposed apo- or Nb3-bound Spike^S2P^ to synchrotron x-ray radiation to label solvent-exposed amino acids with hydroxyl radicals, which we subsequently quantified by mass spectrometry of protease-digested Spike^S2P^ (*18*). Two neighboring surface residues on the S_1_ NTD of Spike (Met^177^ and His^207^) were protected in the presence of Nb3 at a level consistent with prior observations of antibody-antigen interactions by hydroxyl radical footprinting (fig. S5) (*19*). Previously discovered coronavirus neutralizing antibodies bind an epitope within the NTD of Spike with Fab fragments that are noncompetitive with the host cell receptor (*20*, *21*). Further SPR experiments demonstrated that Nb3 can bind Spike^S2P^ simultaneously with monovalent ACE2 (fig. S6). We hypothesized that the multivalent display of Nb3 on the surface of yeast may account for the partial decrease in Spike^S2P^ binding observed in the presence of ACE2-Fc. Indeed, a trivalent construct of Nb3 with 15–amino acid linkers (Nb3-tri) inhibited Spike^S2P^ binding to ACE2 cells with an IC_50_ of 41 nM (fig. S6). How Nb3-tri disrupts Spike-ACE2 interactions remains unclear.

We next tested the neutralization activity of monovalent and trivalent versions of our top class I (Nb6 and Nb11) and class II (Nb3) nanobodies against SARS-CoV-2 pseudotyped lentivirus using a previously described assay (*22*). Nb6 and Nb11 inhibited pseudovirus infection with IC_50_ values of 2.0 and 2.4 μM, respectively. Nb3 inhibited pseudovirus infection with an IC_50_ of 3.9 μM (Fig. 3C and table S1). Nb6-tri shows a 2000-fold enhancement of inhibitory activity, with an IC_50_ of 1.2 nM, whereas trimerization of Nb11 and Nb3 resulted in more modest gains of 40- and 10-fold (51 and 400 nM), respectively (Fig. 3C). We confirmed these neutralization activities with a viral plaque assay using live SARS-CoV-2 virus infection of VeroE6 cells. Here, Nb6-tri proved exceptionally potent, neutralizing SARS-CoV-2 with an average IC_50_ of 160 pM (Fig. 3D). Nb3-tri neutralized SARS-CoV-2 with an average IC_50_ of 140 nM (Fig. 3D).

**Fig. 4:**
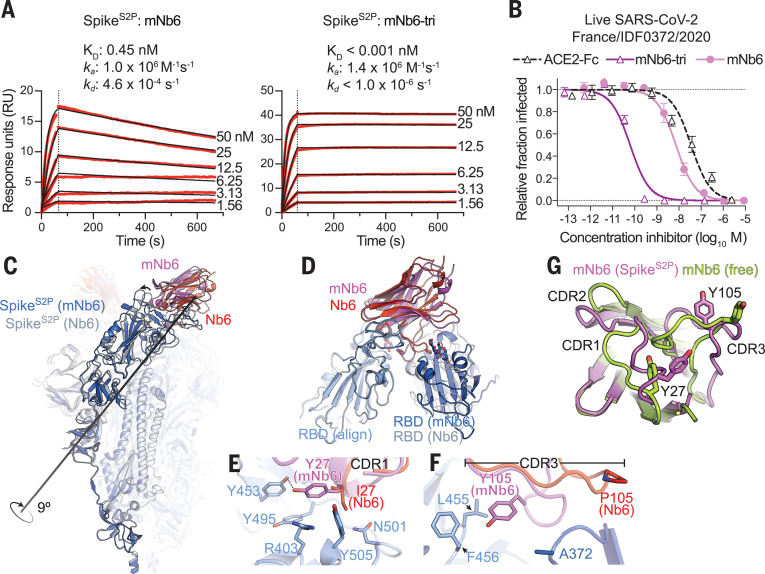
Affinity maturation of Nb6 yields a picomolar SARS-CoV-2 neutralizing molecule. (**A**) SPR of mNb6 and mNb6-tri binding to immobilized Spike^S2P^. Red traces show raw data, and black lines show global kinetic fit. No dissociation was observed for mNb6-tri over 10 min. (**B**) mNb6 and mNb6-tri inhibit SARS-CoV-2 infection of VeroE6 cells in a plaque assay. Representative biological replicate with *n* = 4 technical replicates per concentration. *n* = 3 biological replicates for all samples. All error bars represent SEM. (**C**) Comparison of closed Spike^S2P^ bound to mNb6 and Nb6. Rotational axis for RBD movement is highlighted. (**D**) Comparison of RBD engagement by Nb6 and mNb6. One RBD was used to align both structures (RBD align), demonstrating changes in Nb6 and mNb6 position and the adjacent RBD. (**E**) CDR1 of Nb6 and mNb6 binding to the RBD. As compared to I27 in Nb6, Y27 of mNb6 hydrogen bonds to Y453 and optimizes π-π and π-cation interactions with the RBD. N, Asp; R, Arg. (**F**) CDR3 of Nb6 and mNb6 binding to the RBD demonstrating a large conformational rearrangement of the entire loop in mNb6. A, Ala; L, Leu; F, Phe. (**G**) Comparison of mNb6 complementarity-determining regions in either the cryo-EM structure of the Spike^S2P^-mNb6 complex or an x-ray crystal structure of mNb6 alone.

We further optimized the potency of Nb6 by selecting a saturation mutagenesis library targeting all three CDRs. Two rounds of selection identified high-affinity clones with two penetrant mutations: I27Y (Ile^27^→Tyr) in CDR1 and P105Y (Pro^105^→Tyr) in CDR3. We incorporated these mutations into Nb6 to generate matured Nb6 (mNb6), which binds with 500-fold increased affinity to Spike^S2P^ (Fig. 4A). mNb6 inhibits both pseudovirus and live SARS-CoV-2 infection with low nanomolar potency, a ~200-fold improvement compared with Nb6 (Fig. 4B and table S1).

A 2.9-Å cryo-EM structure shows that mNb6 binds to closed Spike^S2P^ (Fig. 4C and fig. S7). mNb6 induces a slight rearrangement of the down-state RBDs as compared with Spike^S2P^ bound to Nb6, inducing a 9° rotation of the RBD away from the central threefold-symmetry axis. This deviation likely arises from a different interaction between CDR3 and Spike^S2P^, which nudges the RBDs into a new resting position (Fig. 4D). Although the I27Y substitution optimizes local contacts between CDR1 in its original binding site on the RBD, the P105Y substitution leads to a marked rearrangement of CDR3 in mNb6 (Fig. 4, E and F). This conformational change yields a different set of contacts between mNb6 CDR3 and the adjacent RBD. An x-ray crystal structure of mNb6 alone revealed dramatic conformational differences in CDR1 and CDR3 between free and Spike^S2P^-bound mNb6 (Fig. 4G and table S3). Although differences in loop conformation in the crystal structure may arise from crystal lattice contacts, they are suggestive of conformational heterogeneity for unbound mNb6 and induced-fit rearrangements upon binding to Spike^S2P^.

The binding orientation of mNb6 is similar to that of Nb6, suggesting that multivalent design would likewise enhance binding affinity. Unlike Nb6-tri, trivalent mNb6 with a 20–amino acid linker (mNb6-tri) bound to Spike^S2P^ with no observable fast-phase dissociation and no measurable dissociation over 10 minutes, yielding an upper bound for the dissociation rate constant *k*_d_ of 1.0 × 10^−6^ s^−1^ (*t*_1/2_> 8 days) and a *K*_D_ of <1 pM (Fig. 4A). mNb6-tri displays further gains in potency in both pseudovirus and live SARS-CoV-2 infection assays with IC_50_ values of 120 pM (5.0 ng/ml) and 54 pM (2.3 ng/ml), respectively (Fig. 4B and table S1). Given the subpicomolar affinity observed by SPR, it is likely that these viral neutralization potencies reflect the lower limit of the assays. mNb6-tri is therefore an exceptionally potent SARS-CoV-2 neutralizing molecule.

We next tested whether viral neutralization by the class I nanobody mNb6 is potentially synergistic with the class II nanobody Nb3-tri. In pseudovirus neutralization assays, we observed an additive effect when combining Nb3-tri with mNb6 (fig. S8). However, the potency for mNb6 viral neutralization was unchanged with increasing concentrations of Nb3-tri, suggesting minimal synergy between these two nanobodies.

**Fig. 5:**
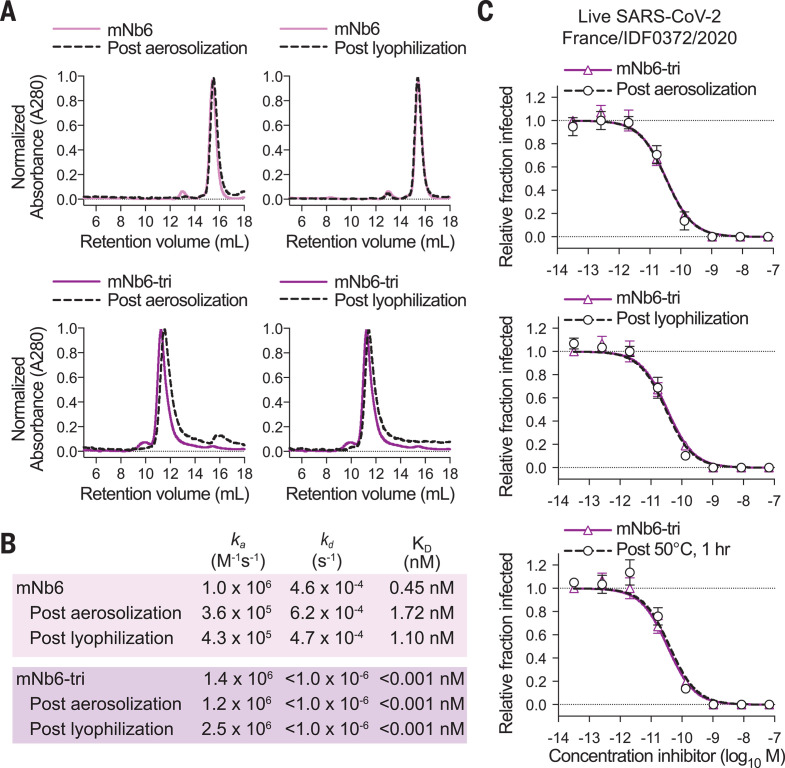
mNb6 and mNb6-tri retain activity after aerosolization, lyophilization, and heat treatment. (**A**) Size exclusion chromatography of nanobodies after lyophilization or aerosolization. (**B**) Summary table of SPR kinetics data and affinities for aerosolized or lyophilized mNb6 and mNb6-tri. (**C**) Inhibition of SARS-CoV-2 infection of VeroE6 cells by mNb6-tri after aerosolization, lyophilization, or heat treatment at 50°C for 1 hour. Representative biological replicate with *n* = 2. Technical replicates are *n* = 3 per concentration.

We next tested Nb6 and its derivatives for stability. Circular dichroism revealed melting temperatures of 66.9°, 62.0°, 67.6°, and 61.4°C for Nb6, Nb6-tri, mNb6, and mNb6-tri, respectively (fig. S9). Moreover, mNb6 and mNb6-tri were stable to lyophilization and to aerosolization, showing no aggregation by size exclusion chromatography, and preserved high-affinity binding to Spike^S2P^ (Fig. 5, A and B, and fig. S9). Finally, mNb6-tri retains potent inhibition of pseudovirus and live SARS-CoV-2 infection after aerosolization, lyophilization, or heat treatment for 1 hour at 50°C (Fig. 5C and fig. S9).

Strategies to prevent SARS-CoV-2 entry into the host cell aim to block the ACE2-RBD interaction. Although high-affinity monoclonal antibodies are leading the way as potential therapeutics (*20*, *23*–*30*), they are expensive to produce by mammalian cell expression and need to be intravenously administered by health care professionals (*31*). Large doses are needed for prophylactic use because only a small fraction of systemic antibodies cross the epithelial cell layers lining the airways (*32*). By contrast, nanobodies can be inexpensively produced in bacteria or yeast. The inherent stability of nanobodies enables aerosolized delivery directly to the nasal and lung epithelia (*33*). Indeed, aerosol delivery of a trimeric nanobody targeting respiratory syncytial virus (ALX-0171) was recently demonstrated to be effective in substantially decreasing measurable viral load in hospitalized infants (*34*). Finally, potential immunogenicity of camelid-derived nanobodies can be mitigated by established humanization strategies (*35*).

Nanobody multimerization has been shown to improve target affinity by avidity (*33*, *36*). In the case of Nb6 and mNb6, structure-guided design of a multimeric construct that simultaneously engages all three RBDs yielded profound gains in potency. Furthermore, because RBDs must be in the up state to engage with ACE2, conformational control of RBD accessibility serves as an added neutralization mechanism (*30*). Indeed, when mNb6-tri engages with Spike, it prevents ACE2 binding both by directly occluding the binding site and by locking the RBDs into an inactive conformation.

Our discovery of class II neutralizing nanobodies demonstrates potentially new mechanisms of disrupting Spike function. The pairing of class I and class II nanobodies in a prophylactic or therapeutic cocktail could provide both potent neutralization and prevention of escape variants (*23*). The combined stability, potency, and diverse epitope engagement of our anti-Spike nanobodies therefore provide a distinctive potential prophylactic and therapeutic strategy to limit the continued toll of the COVID-19 pandemic.

## Supplementary Materials

science.sciencemag.org/content/370/6523/1473/suppl/DC1 Materials and Methods Figs. S1 to S9 Tables S1 to S5 QCRG Structural Biology Consortium Author List References (*37*–*63*) MDAR Reproducibility Checklist

## Appendix

View/request a protocol for this paper from *Bio-protocol*.
